# The Influence of Nicotine, Coffee and Tea on the Digestion

**Published:** 1907-08

**Authors:** 


### THE INFLUENCE OF NICOTINE, COFFEE, AND TEA ON THE DIGESTION.

Cramer, in Muench. Med. Woch., says that in the treatment of diseases of the gastrointestinal tract, it is usually advisable to limit, if not forbid, the use of tobacca, coffee, and tea. In hyperchloridia only very weak coffee should be drank because of its influence in increasing the secretion, and it may be better perhaps to take tea in its place. The use of tobacco should be reduced to a minimum, as smoking appears to excite the secretion of hydrochloric acid and so to increase the hyperchloridia. When there is lack of acidity tea should be forbidden, while coffee may be beneficial in those cases in which the lack of hydrochloric acid in the gastric secretion is not due to irreparable changes in the mucous membrane of the stomach. In motor insufficiency smoking must be reduced to a minimum or completely stopped in all cases in which it is accompanied or followed by an uncomfortable feeling in the stomach. Very little is known in regard to the effect produced by tea and coffee on the motility of the stomach, both seem rather to influence the secretion, but it is certainly an advantage for patients with motor insufficiency to partake of either sparingly. In gastritis a distinction must be made as to whether hyperchloridia or hypochloridia is present, for according to the condition present a moderate use of either coffee or tea may be permitted, but an immoderate use must be controlled in all cases. The same is true in ulcer of the stomach, and the healing of the ulcer will be interfered with if the patient smokes much.—N. Y. Med. Jour. June 29th, 1907.

